# IDH1/MDH1 deacetylation promotes acute liver failure by regulating NETosis

**DOI:** 10.1186/s11658-023-00529-7

**Published:** 2024-01-03

**Authors:** Yukun Wang, Chunxia Shi, Jin Guo, Danmei Zhang, Yanqiong Zhang, Long Zhang, Zuojiong Gong

**Affiliations:** Department of Infectious Diseases, Renmin Hospital of Wuhan University, 238 Jiefang Road, Wuhan, 430060 China

**Keywords:** Deacetylation, Acute liver failure, NETosis

## Abstract

**Background:**

Acute liver failure (ALF) is a life-threatening disease, but its pathogenesis is not fully understood. NETosis is a novel mode of cell death. Although the formation of neutrophil extracellular traps (NETs) has been found in various liver diseases, the specific mechanism by which NETosis regulates the development of ALF is unclear. In this article, we explore the role and mechanism of NETosis in the pathogenesis of ALF.

**Methods:**

Clinically, we evaluated NETs-related markers in the liver and peripheral neutrophils of patients with ALF. In in vitro experiments, HL-60 cells were first induced to differentiate into neutrophil-like cells (dHL-60 cells) with dimethyl sulfoxide (DMSO). NETs were formed by inducing dHL-60 cells with PMA. In in vivo experiments, the ALF model in mice was established with LPS/d-gal, and the release of NETs was detected by immunofluorescence staining and western blotting. Finally, the acetylation levels of IDH1 and MDH1 were detected in dHL-60 cells and liver samples by immunoprecipitation.

**Results:**

Clinically, increased release of NETs in liver tissue was observed in patients with ALF, and NETs formation was detected in neutrophils from patients with liver failure. In dHL-60 cells, mutations at IDH1-K93 and MDH1-K118 deacetylate IDH1 and MDH1, which promotes the formation of NETs. In a mouse model of ALF, deacetylation of IDH1 and MDH1 resulted in NETosis and promoted the progression of acute liver failure.

**Conclusions:**

Deacetylation of IDH1 and MDH1 reduces their activity and promotes the formation of NETs. This change aggravates the progression of acute liver failure.

## Background

Acute liver failure (ALF), characterized by inflammation-mediated liver cell injury, usually presents with acute onset, rapid progression, and high mortality [1]. Various factors, such as hepatitis virus, hepatotoxic drugs, and liver ischemia‒reperfusion injury, can cause liver failure. Although emergency liver transplantation and intensive care management have improved the survival of patients with ALF in recent years [2], the condition of patients with ALF is complicated. Patients show substantial individual differences. At present, no breakthrough has been made in the medical treatment of ALF. Liver failure is a very complex pathophysiological process [3]. Therefore, the pathophysiological mechanism of ALF needs to be further studied to provide a scientific basis for finding new therapeutic targets for ALF.

Studies have shown that various factors, such as viruses, bacteria, activated platelets, cytokines and artificial ingredients (such as phorbol myristate acetate, PMA; lipopolysaccharide, LPS), can activate neutrophils [4–6]. Activated neutrophil nuclei lose their lobulated structure, and neutrophil extracellular traps (NETs) composed of depolymerized chromatin and intracellular granule proteins, that accompany neutrophil death during NETs are formed. This novel inflammatory cell death is called NETosis [4, 5]. NETosis is a specific form of programmed cell death distinct from apoptosis or necrosis [6].

NETosis is divided into two categories: “suicidal” NETosis and “vital” NETosis [7, 8]. “Suicidal” NETosis occurs in the presence of NADPH oxidase activity. When receptors interact with stimuli (e.g., PMA or pathogens), Ca^2+^ is released, leading to protein kinase C (PKC) activation. Subsequently, the NADPH oxidase complex (NOX) is activated, and the levels of reactive oxygen species (ROS) are elevated. This process promotes peptidylarginine deiminase 4 (PAD4) activation, leading to histone citrullination and chromatin depolymerization. Myeloperoxidase (MPO) and neutrophil elastase (NE) translocate to the nucleus. This change promotes the unfolding of chromatin, the rupture of the nuclear envelope, the release of chromatin, granule proteins and other components outside the cell, and the eventual formation and release of NETs [5, 9]. In contrast, “vital” NETosis occurs in the absence of NADPH oxidase activity. “Vital” NETosis is activated by platelets (PLTs), microbes, and complement proteins. After neutrophil activation, Ca^2+^ is transferred into neutrophils through small conductance potassium channel member 3 (SK3). The PAD4 enzyme is activated, leading to citrullinated histone H3 (CitH3) and chromatin decondensation. Finally, NETs are emitted from neutrophils via vesicles. In “vital” NETosis, neutrophils still retain their viability and their phagocytic, chemotaxis, and pathogen-killing functions [7, 8].

Previous studies have shown that NETs were detected in the liver tissue of patients with ALF [10] or mouse models [11]. The levels of NETs markers in the plasma of patients with ALF are correlated with prognosis [10]. Nonetheless, the underlying mechanisms of NETosis in the onset and progression of ALF need further exploration.

The hepatic tricarboxylic acid (TCA) cycle is central to the integrated metabolism of macronutrients (carbohydrates, fatty acids, and amino acids) and is essential for aerobic metabolism. Some scholars have proposed that abnormal liver energy metabolism is involved in the entire pathogenesis [12]. Previous studies have shown that the TCA cycle is disturbed in liver failure models and that an unbalanced TCA cycle leads to reduced oxidative metabolism [13–15]. In our previous study, liquid chromatography with tandem mass spectrometry (LC‒MS/MS) analysis was used to establish the molecular expression profiles in mice with liver failure that were treated with histone deacetylase inhibitor 6 (HDAC6i, ACY1215). The results of quantitative protein sequencing showed that the expression of IDH1 and MDH1 in TCA was different. The expression levels of IDH1 and MDH1 were significantly decreased in the liver failure group compared with the normal group. However, ACY-1215 increased the expression of IDH1 and MDH1 [16]. Our previous studies found that the activities of IDH1 and MDH1 in patients with ALF were significantly reduced by extracting neutrophils from patients with ALF and healthy subjects to prepare cell homogenates. HDAC6i modifies IDH1 at lysine93 (K93) and MDH1 at lysine118 (K118) by acetylation. This process promoted oxidative phosphorylation to alleviate ALF. Therefore, we speculate that deacetylation of IDH1 and MDH1 may promote acute liver failure by regulating NETosis.

## Methods

### Cell lines and human tissue specimens

The HL-60 cell line is a human promyelocytic leukemia cell line (cat. no. GDC0028) and was purchased from the China Type Culture Collection (CTCC). The HL-60 cell line was authenticated using short tandem repeat profiling and tested for mycoplasma infection.

Tissue samples from liver transplant recipients and donors were fixed with 10% neutral formalin, dehydrated and embedded in paraffin, and then the liver tissues were serially sectioned with a paraffin slicer. All patients provided written informed consent. The Research Ethics Committee of Renmin Hospital of Wuhan University approved this study. The human experiment ethics number was WDRY-2021-K016 (approved 9 February 2021).

### Cell culture and DMSO-induced differentiation

The HL-60 cell line was cultured in IMDM (HyClone) with 10% fetal bovine serum (FBS, Beyotime) at 37 °C in a cell incubator containing 5% carbon dioxide. For differentiation of HL-60 cells into neutrophil-like cells (dHL-60), HL-60 cells were cultured with 1.25% DMSO for 1, 3, 5 and 7 days. HL-60 cells cultured in a DMSO-free medium served as a negative control. In the ACY-1215 group, dHL-60 cells were treated with ACY-1215 (cat. HY-16026, MedChemExpress, 10 μmol/l) for 2 h.

### HL-60 cell differentiation detection by flow cytometry

The upregulation of CD11b expression on HL-60 cells is an important feature of the differentiation of promyelocytes into mature granulocytes. DMSO affects the surface expression of CD11b on HL-60 cells. HL-60 cells labeled with CD11b antibody were screened by flow cytometry. HL-60 cells (100 μl) with a cell density of 6–10 × 10^6^ cells/mL in each experimental group were added to 5 μl of PE antihuman CD11b antibody (cat. no. 50-0118-T025, Tonbo Biosciences) and incubated at 37 °C in the dark for 60 min. After centrifugation, the cells were resuspended in phosphate-buffered saline (PBS) and detected by flow cytometry.

### Assessment of nuclear morphology

On days 1, 3, 5, and 7, cells were collected, and differentiated nuclear morphology was assessed by Giemsa staining. Slides were coated with poly-l-lysine (PLL) in advance. Ten microliters of cells at a cell density of 3–5 × 10^5^ cells/ml from each experimental group was spread onto PLL slides. After Giemsa staining, the cells were observed with a microscope. Cell images were randomly taken under a 100× objective lens to observe cell morphology (Olympus, Tokyo, Japan).

### Human neutrophil isolation

Human neutrophil isolation buffer (5 ml) was added to a 15 ml centrifuge tube. Five milliliters of venous blood from patients with ALF or healthy subjects was slowly added to the upper layer of the separation solution and centrifuged at room temperature at a speed of 900*g* for 25 min. After the neutrophil layer was aspirated, red blood cell lysate was added to lyse at 37 °C for 2 min and centrifuged at 900*g* for 5 min to remove residual red blood cells. Two milliliters of IMDM containing 10% FBS was used to resuspend neutrophils.

### PMA-induced NETs formation assay

dHL-60 cells (1 × 10^6^ cells/ml) were inoculated into 24-well plates containing PLL-coated slides and cultured in a cell culture incubator for 30 min. Cells in the experimental group were stimulated with 100 ng/mL PMA (Cat. HY-18739, MedChemExpress) for 4 h. Moreover, cells were incubated with IMDM containing 10% FBS to serve as a negative control. Cells were blocked with 10% BSA for 30 min at 37 °C for immunofluorescence staining.

### Mouse

Male 5–6-week-old C57BL/6 J wild-type mice were purchased from Wuhan Biomedical Research Institute of Wuhan University. They were raised in the animal facility of Renmin Hospital of Wuhan University under a light/dark cycle of 12/12 h, constant temperature (25 °C), and constant humidity (55 ± 5%). The Animal Care and Use Committee of Renmin Hospital of Wuhan University, China approved the experiments [approval number WDRM (Welfare) 20181018; approved on 26 October 2018].

### In vivo experiment

The mice were randomly divided into six groups with six mice in each group: control group: normal saline; ALF model group: lipopolysaccharide (LPS)/d-galactosamine (d-gal); ACY1215 group: LPS/d-gal + ACY1215; IDHI-K93R group: IDHI-K93R adenovirus-infected mice + LPS/d-gal; MDHI-K118R group: MDHI-K118R adenovirus-infected mice + LPS/d-gal; empty group: empty adenovirus + LPS/d-gal. LPS (100 μg/kg; Sigma-Aldrich) combined with d-gal (400 mg/kg) was injected intraperitoneally [17, 18]. All mice were modeled by intraperitoneal injection. Mice in the control group were given an equal volume of normal saline. For the ALF model group, d-gal combined with LPS was administered. In the ACY1215 group, ACY1215 (25 mg/kg) [19] was intraperitoneally injected 2 h before d-gal/LPS administration. The IDHI-K93R/MDH1-K118R/empty adenovirus group received an intraperitoneal injection of LPS/d-gal 1 month after tail vein injection of adenovirus. Twenty-four hours after LPS/d-gal administration, animals were quickly euthanized by CO_2_ inhalation, and blood samples and liver tissues were subsequently collected. All animal studies, including mouse euthanasia procedures, were performed in accordance with the regulations of the Animal Care and Use Committee of Renmin Hospital of Han University.

### Cell transfection or animal infection

In vitro experiments, the endotoxin-free plasmid mini-prep kit (cat. DP118; Tiangen) was used to extract plasmids from amplified *Escherichia coli*. Hieff Trans™ Suspension Cell-Free Lipofectamine Reagent (cat. 40805ES03; Yeasen) was used for transient transfection. IDH1-WT, IDH1K93R, MDH1-WT, MDH1K118R, and blank control plasmids were transfected into dHL-60 cells at 30 µg/ml. Adenoviral vectors expressing IDH1K93R and MDH1K118R and empty vector were injected into the tail vein for in vivo experiments.

### Hematoxylin–eosin staining (HE staining)

Liver tissue sections were evaluated by HE staining for pathological changes in liver tissue. The degree of liver injury in the ALF model was assessed by light microscopy (Olympus, Tokyo, Japan) scoring.

### Western blot analysis

Liver tissue and cells were lysed using RIPA lysis buffer (Beyotime) containing cocktail (Beyotime). Lysates were sonicated and centrifuged at 12,000*g* for 10 min at 4 °C. A total of 30 μg of protein was loaded onto sodium dodecyl sulfate‒polyacrylamide gel electrophoresis gel loading wells, and polyacrylamide gels were prepared and transferred to polyvinylidene fluoride membranes. Membranes were blocked in 5% BSA and incubated with primary and secondary antibodies. Proteins were detected using an enhanced chemiluminescence system (cat. BL523A, Biosharp), and ImageLab statistical software (Bio-Rad, Hercules, CA) was used to evaluate band intensities on western blots. The following primary antibodies were used: anti-PAD4 (cat. no. 17373-1-AP, Proteintech) and antihistone H3 (citrulline R2 + R8 + R17) (ab5103, Abcam).

### Immunoprecipitation assay (IP)

RIPA lysate/RIPA lysis buffer and antiacetylation antibody cocktail (100:1:1) were added to lyse liver tissue and cells. Lysates were sonicated and centrifuged at 12,000*g* for 10 min at 4 °C. Some proteins were removed as input groups. The remaining proteins were incubated overnight at 4 °C with anti-MDH1 antibody (cat. no. 66505–1-Ig, Proteintech) or anti-IDH1 antibody (cat. no. 66197–1-Ig, Proteintech). Then, 20 µl of precleaned protein A/G-agarose (cat. P2197S; Beyotime) was added per 100 µl of protein lysate for 4 h at 4 °C. After centrifugation, the precipitate was collected and added to 20 µl of SDS‒PAGE sample loading buffer (1X) and heated at 100 °C for 5 min. Anti-IDH1 (cat. no. 12332-1-AP, Proteintech), anti-MDH1 (cat. no. 15904-1-AP, Proteintech) or anti-acetyl-lysine (cat. 9441; Cell Signaling Technology) was used for verification by western blotting. IDH1 antibody and MDH1 antibody were used as target antibodies. A mouse monoclonal antibody IgG1 isotype control was used as a control antibody.

### Immunofluorescence staining

Neutrophils were isolated, and after PMA induction, dHL-60 cells were adhered to PLL-pretreated sterile glass slides. Liver tissue sections were fixed on glass slides. Four percent paraformaldehyde was used to fix the cells for 30 min. Then, 50–100 μl of 2% permeabilization working solution was added to the cells. The sections were blocked with 10% bovine serum albumin for 30 min. Goat anti-human/mouse myeloperoxidase antibody (cat. no. AF3667, Novus Biologicals) and rabbit anti-histone H3 (citrulline R2 + R8 + R17) antibody (cat. no. ab5103, Abcam) were diluted 1:100, and slides were incubated overnight at 4 °C in a humid chamber. Then, the slides were incubated with secondary antibodies (1:200 dilution, Thermo Fisher Scientific, cat. A32814 and A10042) for 1 h at room temperature. Then, they were stained with 4′,6-diamidino-2-phenylindole (DAPI) for 5 min. They were imaged using a fluorescence microscope (Olympus). In liver tissues, five microscopic fields per liver section were counted using ImageJ analysis software. The average fluorescence intensity of Cit-H3 and MPO was quantified [20]. Among cells, the percentage of NETs was calculated as the average of five fields normalized to the total number of neutrophils or dHL-60 cells, and the results are expressed as the mean ± standard deviation (SD)[21].

### Quantitative experiment of cfDNA in NETs

Studies have shown that soluble NETs residues, in the form of cell free DNA (cfDNA), exist in supernatants in vitro and serum or tissue fluids in vivo, and it can be detected by PicoGreen[22]. The cfDNA content in NETs was detected using the Quant-It™ PicoGreen^®^ dsDNA kit (cat. P7581, Invitrogen) according to the instructions of the kit [23]. Lambda DNA standard (100 μg/ml) from the kit was diluted with 1× TE buffer for standard DNA preparation, and 0.1, 1, 10, 100, and 1000 ng/ml were used for the different concentrations. Different samples and standards were added to the plate, with three wells for each sample and 100 μl of samples or standards in each well. A total of 100 μl of Quant-It PicoGreen working solution was added to each well, mixed thoroughly, and incubated at room temperature in the dark for 5 min. After incubation, a multifunctional microplate reader (excitation light of 490 nm and emission light of 520 nm) was used for detection. We calculated the sample concentration in each well based on the known concentrations of standard substances and the measured fluorescence intensity and then measured the fluorescence to obtain a standard curve. The fluorescence obtained from the sample was compared with the standard curve and expressed in ng/ml.

### Detection of cell apoptosis by the terminal deoxynucleotidyl transferase dUTP nick-end labeling (TUNEL) method

The TUNEL method was used to detect apoptosis in liver samples. The liver tissue sections were washed, diluted, digested, labeled, and stained with DAB and counterstained with hematoxylin. The sample was observed under an optical microscope (Olympus), and apoptotic bodies (brown granules) in the nucleus indicated cell positivity.

### Statistical analysis

All results are expressed as the mean ± standard deviation and were analyzed using GraphPad Prism 9 (San Diego, CA, USA). Differences between groups were assessed using two-tailed unpaired Student’s *t* test or one-way analysis of variance (ANOVA), depending on the condition. *P* values less than 0.05 were considered statistically significant.

## Results

### Increased formation of NETs in patients with acute liver failure

**Fig. 1:**
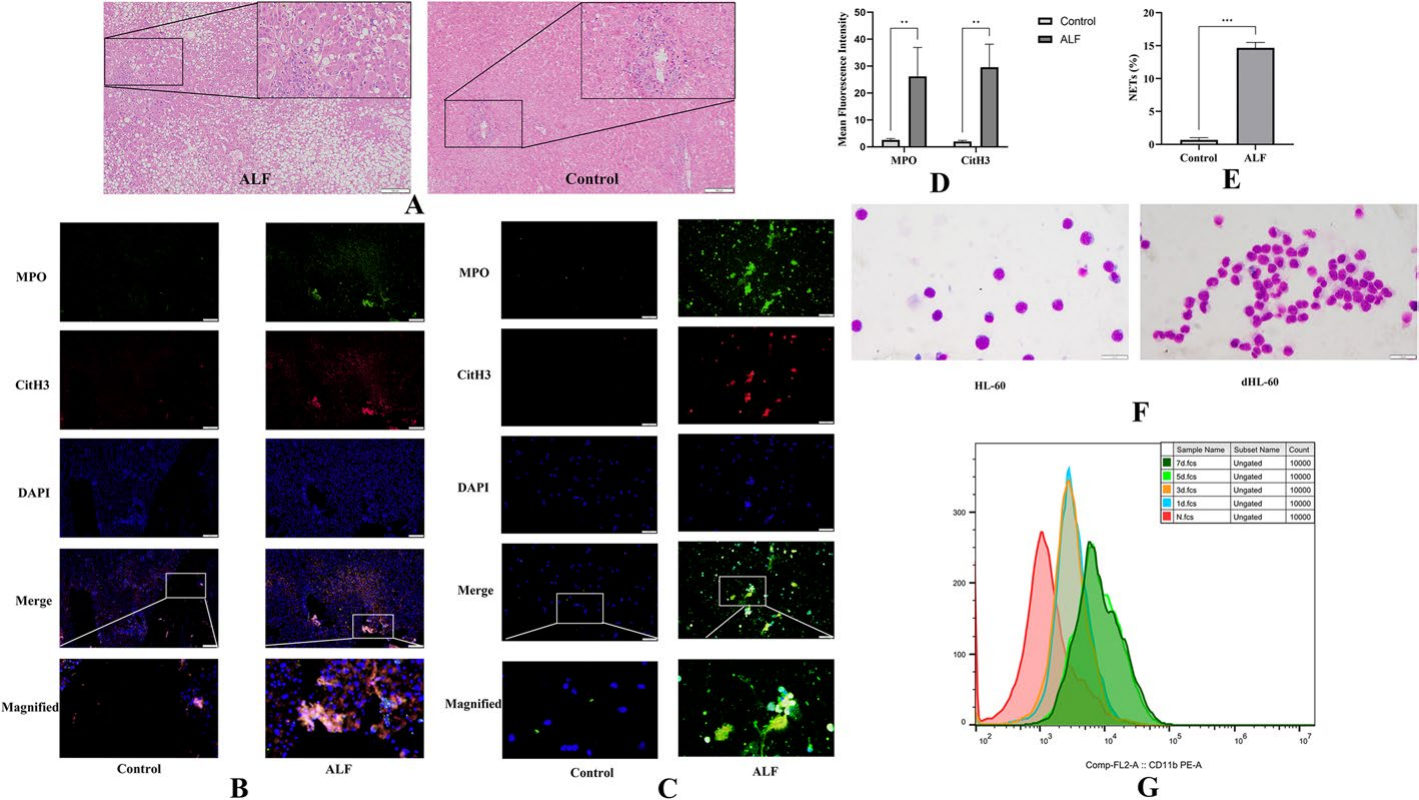
Increased formation of NETs in patients with acute liver failure and differentiation of HL-60 cells in the presence of DMSO. **A** HE staining of liver samples in the healthy controls and patients with acute liver failure; the scale bar is 100 μm. **B** Liver samples from healthy controls and patients with acute liver failure were assessed by immunofluorescence staining with MPO (green), CitH3 (red), and DAPI (blue) to detect the formation of NETs. The scale bar is 50 μm. **C** Representative immunofluorescence images showing the formation of NETs from neutrophils of the healthy controls and patients with ALF. NETs formation detected by MPO (green), CitH3 (red), and DAPI (blue); the scale bar 50 μm. **D** The mean fluorescence intensity of Cit-H3 and MPO was quantified using ImageJ analysis software. Five microscopic fields per liver section were counted. **E** NETs and neutrophils were counted after immunofluorescence staining with MPO and CitH3. The percentage of NETs was calculated as the average of five fields normalized to the total number of neutrophils. **F** HL-60 and HL-60 cells were treated with 1.25% DMSO for 5 days (dHL-60). Cell morphology was shown by Giemsa staining; the scale bar is 10 μm. **G** CD11b expression was used as a neutrophil surface marker to measure the differentiation status of HL-60 cells on days 0, 1, 3, 5, and 7. *NETs* Neutrophil extracellular traps, *MPO* Myeloperoxidase, *CitH3* Citrullinated histone H3, *DAPI* 4′,6-diamidino-2-phenylindole. The histograms show the means ± SD, **P < 0.01, ***P < 0.001

HE staining showed the liver structure in each group. Compared with healthy controls, patients with acute liver failure had extensive death of hepatocytes and significantly increased numbers of infiltrating inflammatory cells (Fig. 1A). The formation of NETs was shown by the increased colocalization of CitH3 and MPO [20]. We observed increased formation of NETs in patients with acute liver failure compared with healthy individuals (Fig. 1B). NETs were quantified by the mean fluorescence intensity of Cit-H3 and MPO (Fig. 1D). We further assessed the ability of neutrophils from patients with ALF and healthy individuals to produce NETs. Immunofluorescence revealed that neutrophils in the plasma of the patients with ALF were more likely to form NETs than those in the plasma of the healthy controls, which produced few of these structures. The neutrophils in the ALF group could release MPO (green) and CitH3 (red) and release them out of the cells simultaneously with DNA (blue), forming an irregular cord-like fiber network (Fig. 1C, E).

### Analysis of the mechanism of NETs in neutrophil-like HL-60 cells (dHL-60 cells)

Neutrophils are terminally differentiated cells that cannot be genetically modified. It is thus difficult to elucidate the role of different proteins in the different stages of NETosis. These cells have a short lifespan, which also limits their use. Therefore, differentiated HL-60 cells (dHL-60) have been used to study the mechanism of NETs formation [24]. Giemsa staining also showed the same result. Most of the induced HL-60 cells successfully differentiated into neutrophil-like cells (dHL-60) after 5 days (Fig. 1F). CD11b was used as a neutrophil surface marker to assess cell differentiation efficiency [24]. Flow cytometry showed that CD11b was effectively expressed in dHL-60 cells from day 5 induced by 1.25% DMSO (Fig. 1G). Therefore, dHL-60 cells differentiated with 1.25% DMSO for 5 days were used as model cells of neutrophils to study the mechanism of NETs formation.

### The IDH1-K93R and MDH1-K118R mutations resulted in reduced acetylation levels

**Fig. 2:**
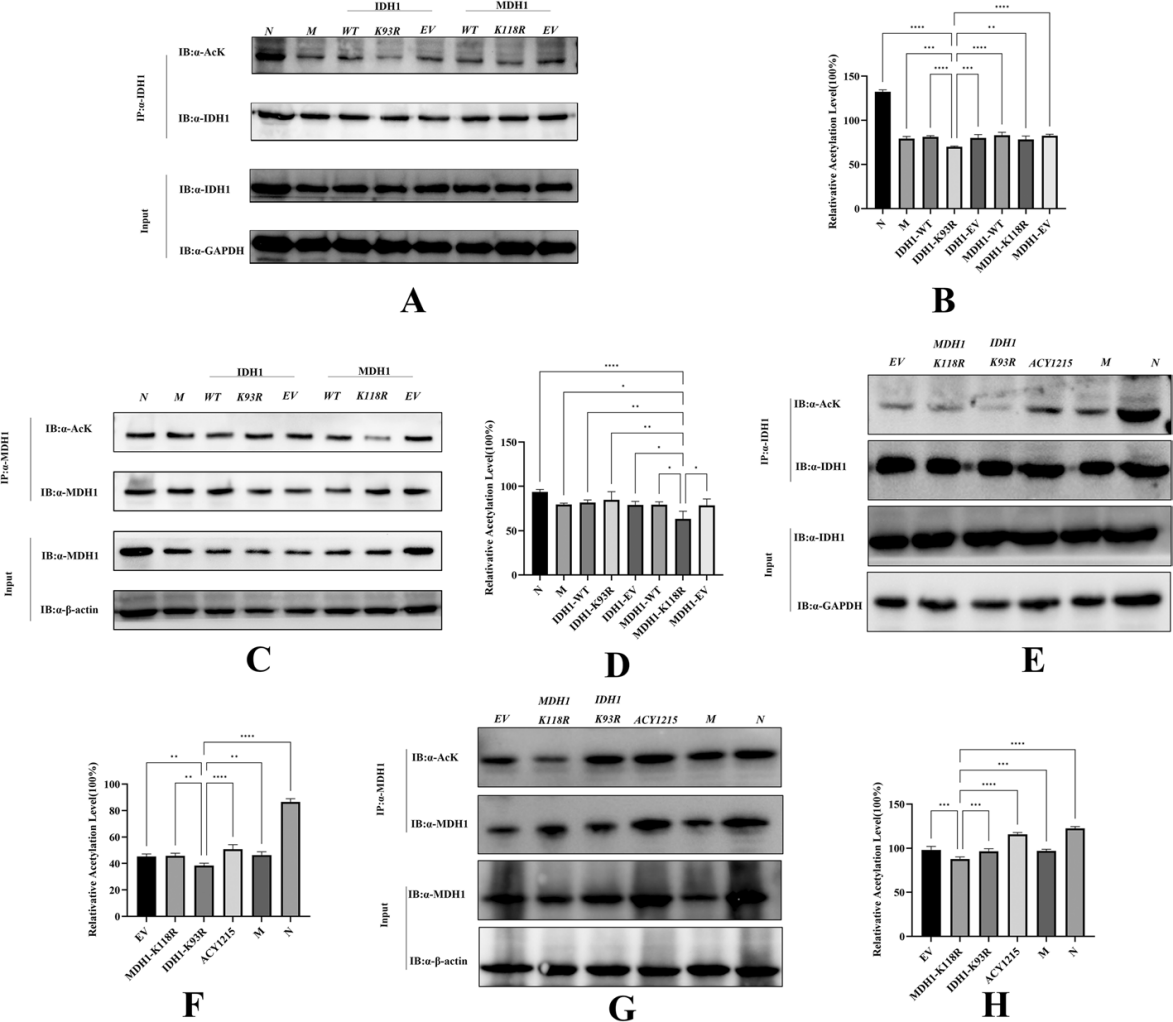
Mutation of IDH1-K93R and MDH1-K118R resulted in reduced acetylation levels. **A** dHL-60 cells were analyzed by western blotting using a pan-antiacetyl lysine antibody to detect the acetylation level of IDH1 purified by the IDH1 antibody. **B** Relative ratios of IDH1 acetylation were calculated after normalization to IDH1. **C** dHL-60 cells were analyzed by western blotting using a pan‐antiacetyl lysine antibody to detect the acetylation level of MDH1 purified by the MDH1 antibody. **D** Relative ratios of MDH1 acetylation were calculated after normalization to MDH1. (N: dHL-60; M: dHL-60 + PMA; IDH1-WT: IDH1-wild-type + PMA; IDH1-K93R:IDH1-K93R + PMA; IDH1-EV: IDH1-Flag + PMA; MDH1-WT: MDH1-wild-type + PMA; MDH1-K118R:MDH1-K118R + PMA; MDH1-EV: MDH1-HA + PMA.) **E** Liver samples were analyzed by western blotting using a pan‐antiacetyl lysine antibody to detect the acetylation level of IDH1 purified by IDH1 antibody. **F** Calculation of the relative ratio of IDH1 acetylation after normalization to IDH1. **G** Liver samples were analyzed by western blotting using a pan‐antiacetyl lysine antibody to detect the acetylation level of MDH1 purified from the MDH1 antibody. **H** Calculation of relative ratios of MDH1 acetylation after normalization to MDH1. N: liver samples injected with normal saline; M: ALF model, liver samples injected with LPS/d-gal; ACY1215: LPS/d-gal + ACY1215; IDHI-K93R: IDHI-K93R adenovirus-infected mice + LPS/d-gal; MDHI-K118R: MDHI-K118R adenovirus-infected mice + LPS/d-gal group, EV: empty adenovirus-infected mice + LPS/d-gal. The histograms show the means ± SD. *P < 0.05, **P < 0.01, ***P < 0.001, and ****P < 0.0001

In dHL-60 cells, after mutation of the K118 of the MDH1 plasmid and the K93 of IDH1, the amount of acetylated lysine bound by IDH1 or MDH1 was detected by immunoprecipitation. IDH1-K93R or MDH1-K118R showed significantly reduced acetylation levels compared with the other groups (Fig. 2A–D). The amount of acetylated lysine bound by IDH1 or MDH1 in mouse liver was detected by immunoprecipitation. Compared with other groups, the IDH1-K93R adenovirus group or the MDH1-K118R adenovirus group showed a significant reduction in acetylation levels (Fig. 2E–H).

### IDH1/MDH1 deacetylation enhanced NETs formation

**Fig. 3:**
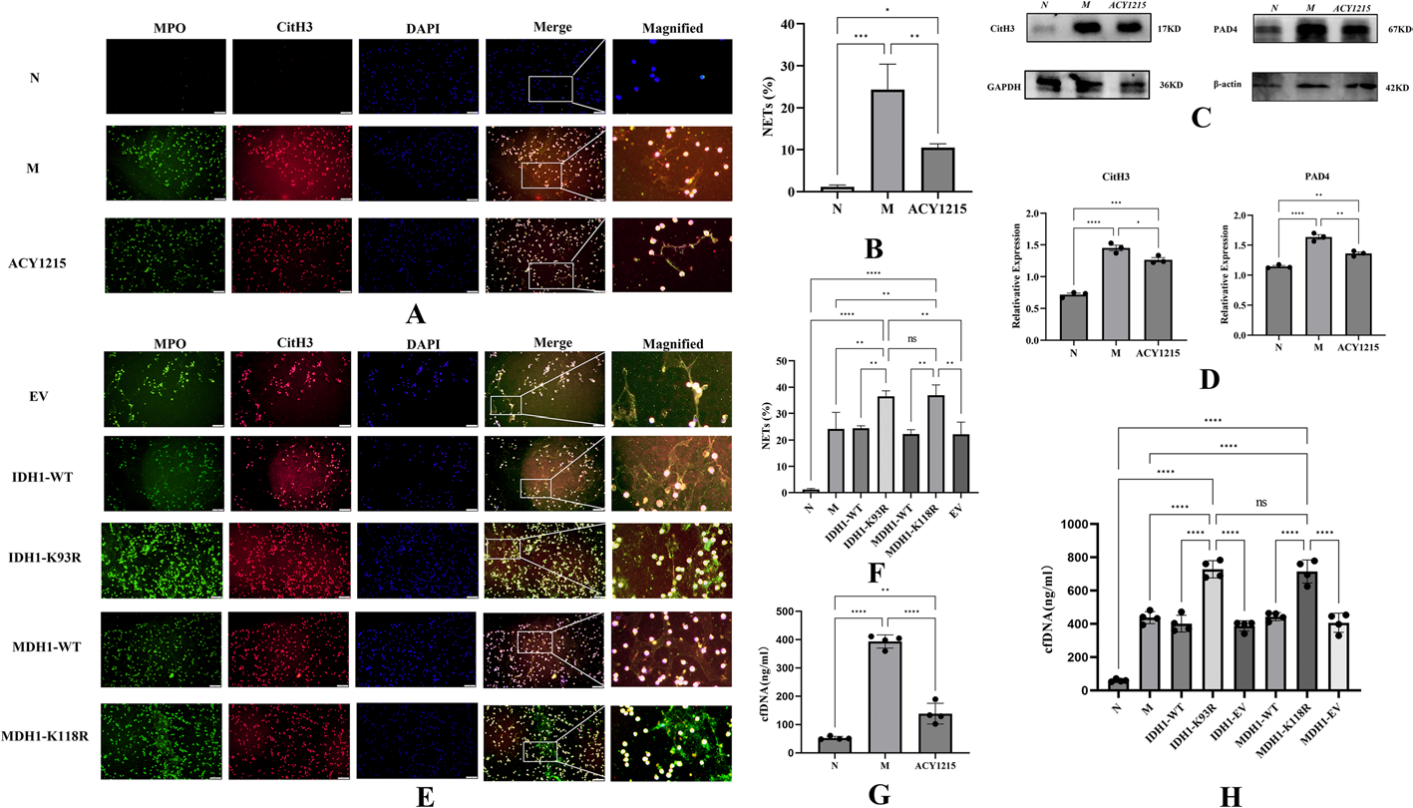
IDH1/MDH1 deacetylation enhanced NETs formation. **A** dHL-60 cells were stimulated with PMA in vitro, left untreated/treated with ACY1215, and immunofluorescently stained with MPO (green), CitH3 (red), and DAPI (blue) to detect the formation of NETs; the scale bar is 50 μm. **B** NETs and dHL-60 cells were counted after immunofluorescence staining with MPO and CitH3. The percentage of NETs was calculated as the average of five fields normalized to the total number of dHL-60 cells. **C** In dHL-60 cells, the protein levels of CitH3 and PAD4 were measured by western blotting. **D** In dHL-60 cells, the protein levels of CitH3 and PAD4 were analyzed by ImageLab. **E** In dHL-60 cells, with plasmid mutations at K118 of MDH1 and K93 of IDH1, cells were stimulated with PMA in vitro, and immunofluorescence staining with MPO (green), CitH3 (red), and DAPI (blue) was performed to detect the formation of NETs; the scale bar is 50 μm. **F** NETs and dHL-60 were counted after immunofluorescence staining with MPO and CitH3 to calculate the proportion of dHL-60 releasing NETs. **G** NETs were quantified by a Quant-iT™ PicoGreen^®^ dsDNA kit. **H** NETs were quantified by a Quant-iT™ PicoGreen^®^ dsDNA kit. N:dHL-60; M: dHL-60 + PMA; ACY1215: dHL-60 + PMA + ACY1215;IDH1-WT: IDH1-wild-type + PMA; IDH1-K93R:IDH1-K93R + PMA; IDH1-EV: IDH1-Flag + PMA; MDH1-WT: MDH1-wild-type + PMA; MDH1-K118R:MDH1-K118R + PMA; MDH1-EV: MDH1-HA + PMA, *NETs* Neutrophil extracellular traps, *cfDNA* Cell-free DNA; *MPO* Myeloperoxidase, *CitH3* Citrullinated histone H3, *DAPI* 4′,6-diamidino-2-phenylindole, *PAD4* peptidylarginine deiminase 4.The histograms show the means ± SD. *ns* no significance, *P < 0.05, **P < 0.01, ***P < 0.001, and ****P < 0.0001

We assessed NETs formation by observing the release of MPO (green) and CitH3 (red), which form irregular cord-like fiber networks with DNA (blue). After 4 h of stimulation with 100 ng/ml PMA, a significant increase in the formation of NETs was observed compared with that of the control group (Fig. 3A, B). However, the ability of dHL-60 cells to form NETs was significantly reduced after ACY1215 treatment (Fig. 3A, B). As a key enzyme of NETs, PAD4 mediates chromatin decondensation and NETs formation by histone hypercitrullination [25]. After ACY1215 treatment, western blotting showed that the expression levels of CitH3 and PAD4 were decreased compared with those in the PMA-stimulated group (Fig. 3C, D). Furthermore, we quantified NETs by PicoGreen [23] and showed significantly reduced NETs release after ACY1215 treatment (Fig. 3G). Further studies revealed significantly increased NETs formation upon deacetylation of IDH1/MDH1 compared with the wild-type (WT) controls (3E and 3F). Meanwhile, we quantified NETs by PicoGreen and showed significantly increased NETs release in the IDH1-K93R and MDH1-K118R groups compared with the WT control group (Fig. 3H). Furthermore, western blotting analysis showed that the expression levels of CitH3 and PAD4 were significantly increased compared with those in the WT controls (4A–4D).

### IDH1/MDH1 deacetylation enhanced NETs formation and exacerbated LPS/d-gal-induced liver injury in mice

**Fig. 4:**
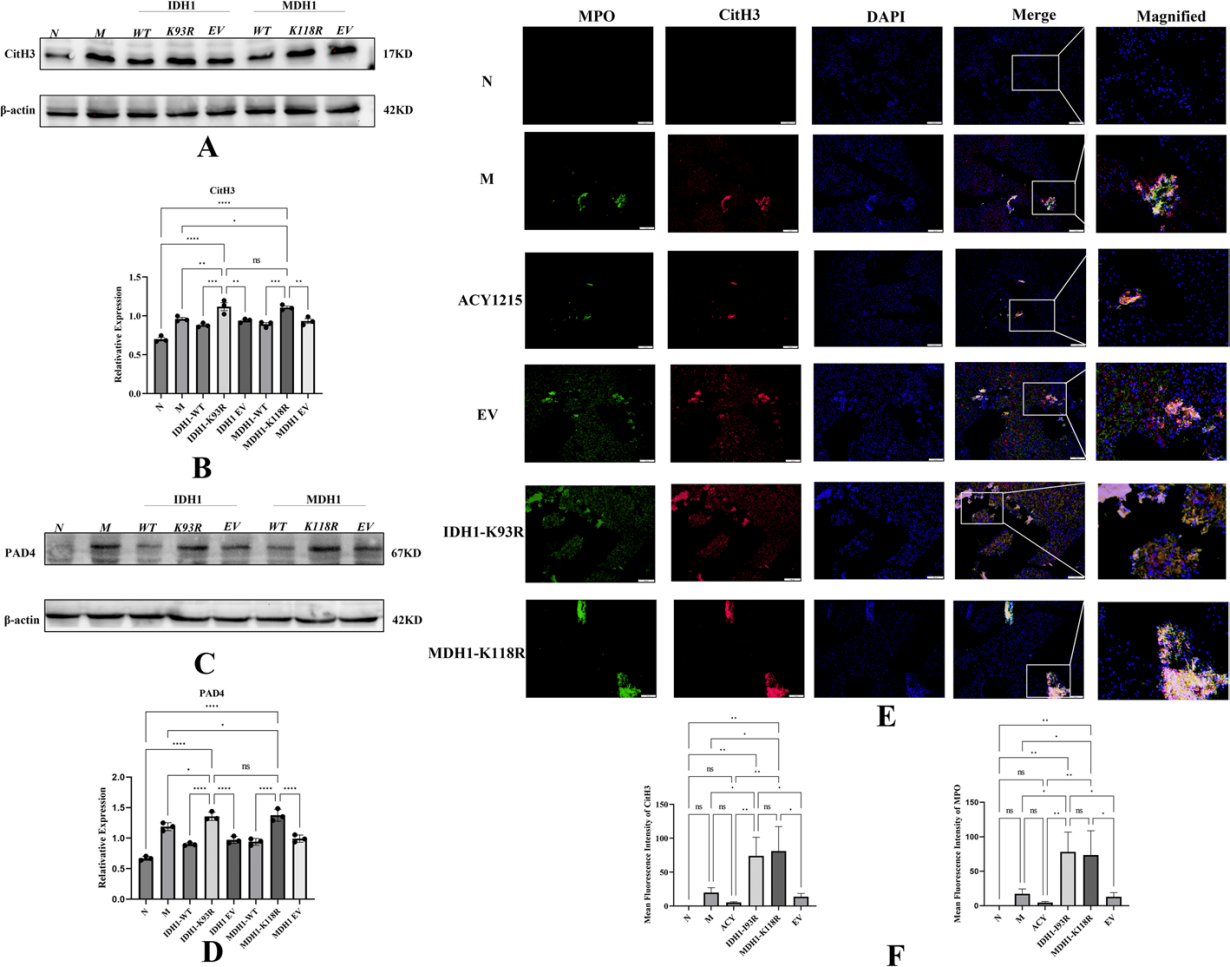
IDH1/MDH1 deacetylation enhanced NETs formation in mice with LPS/d-gal-induced liver injury. **A**, **B** In dHL-60 cells, with plasmid mutations at the K118 site of MDH1 and the K93 site of IDH1, cells were stimulated with PMA in vitro, and the expression of CitH3 was measured by western blotting and analyzed by ImageLab. **C**, **D** In dHL-60 cells, the expression of PAD4 was measured by western blotting and analyzed by ImageLab (N: dHL-60; M: dHL-60 + PMA; IDH1-WT: IDH1-wild-type + PMA; IDH1-K93R: IDH1-K93R + PMA; IDH1-EV: IDH1-Flag + PMA; MDH1-WT: MDH1-wild-type + PMA; MDH1-K118R:MDH1-K118R + PMA; MDH1-EV: MDH1-HA + PMA.). **E** Liver samples were detected for NETs by immunofluorescence staining with MPO (green), CitH3 (red), and DAPI (blue); the scale bar is 50 μm. **F** The mean fluorescence intensity of Cit-H3 and MPO was quantified using ImageJ analysis software. Five microscopic fields per liver section were counted. N: liver samples injected with normal saline; M: ALF model, liver samples injected with LPS/d-gal; ACY1215: LPS/d-gal + ACY1215; IDHI-K93R: IDHI-K93R: adenovirus-infected mice + LPS/d-gal; MDHI-K118R: MDHI-K118R adenovirus-infected mice + LPS/d-gal group; EV: empty adenovirus-infected mice + LPS/d-gal, *MPO* Myeloperoxidase, *CitH3* Citrullinated histone H3, *DAPI* 4′,6-diamidino-2-phenylindole, *PAD4* peptidylarginine deiminase 4, *ns* no significance. The histograms show the means ± SD. *P < 0.05, **P < 0.01, ***P < 0.001, and ****P < 0.0001

**Fig. 5:**
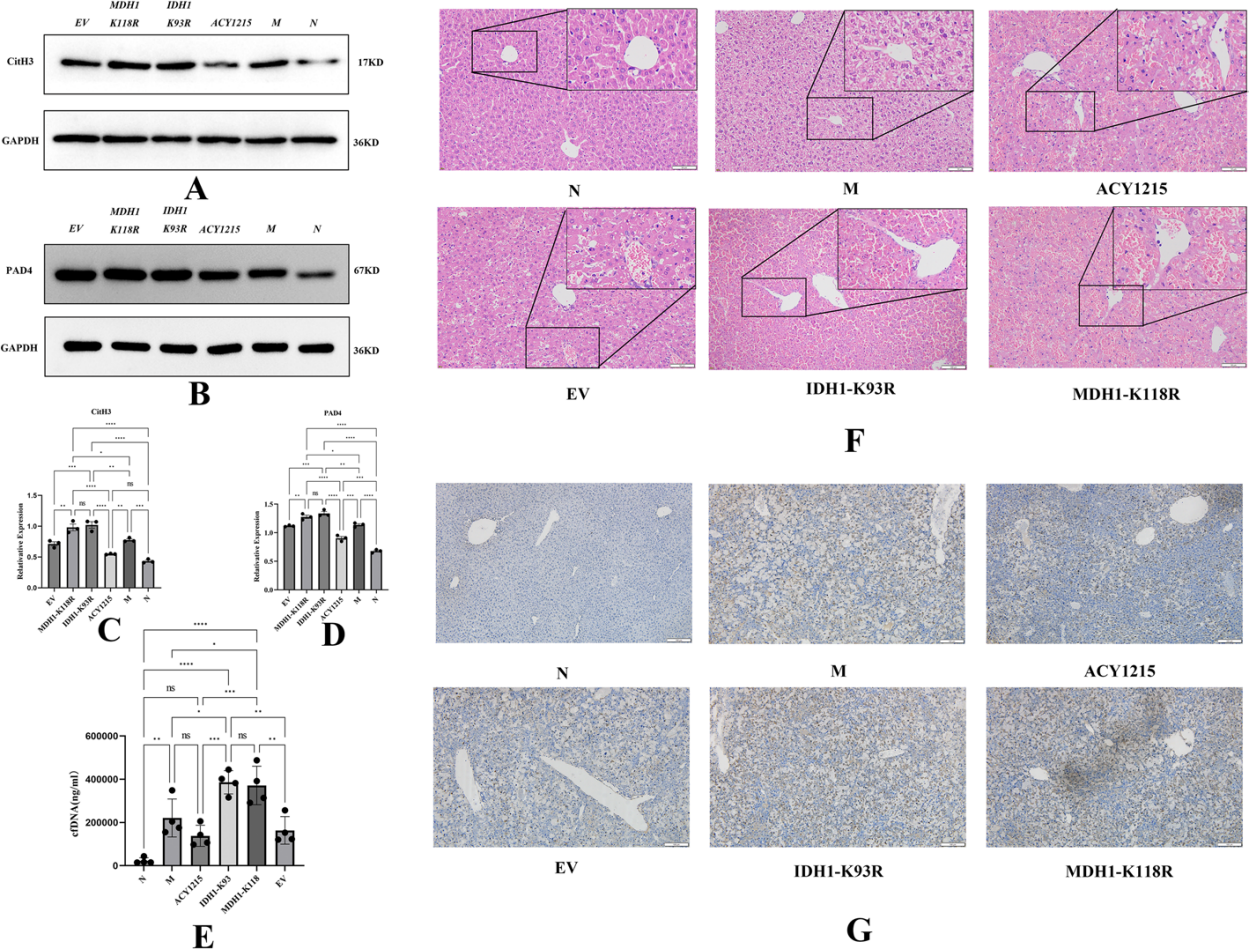
IDH1/MDH deacetylation promotes acute liver failure by regulating NETosis. **A**, **C** The expression of hepatic CitH3 was measured by western blotting and analyzed by ImageLab. **B**, **D** The expression of hepatic PAD4 was measured by Western blotting and analyzed by ImageLab. **E** NETs were quantified by a Quant-iT™ PicoGreen^®^ dsDNA kit. **F** HE staining of mouse liver tissue under different treatments; the scale bar is 100 μm. **G** Cell apoptosis was detected by the TUNEL method in the liver tissue of mice under different treatments; the scale bar is 100 μm. N: liver samples injected with normal saline; M: ALF model, liver samples injected with LPS/d-gal; ACY1215: LPS/d-gal + ACY1215; IDHI-K93R: IDHI-K93R adenovirus-infected mice + LPS/d-gal; MDHI-K118R: MDHI-K118R adenovirus-infected mice + LPS/d-gal group; EV: empty adenovirus-infected mice + LPS/d-gal, *CitH3* citrullinated histone H3, *PAD4* peptidylarginine deiminase 4, *cfDNA* Cell-free DNA. The histograms show the means ± SD. *ns* no significance, *P < 0.05, **P < 0.01, ***P < 0.001, and ****P < 0.0001. *P < 0.05, **P < 0.01, ***P < 0.001, and ****P < 0.0001

Immunofluorescence showed that the hepatic colocalization of CitH3 and MPO was reduced in the ACY1215 group compared with the ALF model group. However, hepatic colocalization of CitH3 and MPO was significantly increased in the IDH1-K93R or MDH1-K118R mice compared with the other groups (Fig. 4E). NETs were quantified by the mean fluorescence intensity of Cit-H3 and MPO (Fig. 4F). This finding suggests increased NETs formation upon deacetylation of IDH1/MDH1. Similar conclusions were obtained by western blotting. The levels of the NETs markers CitH3 and PAD4 were significantly increased in the IDH1-K93R and MDH1-K118R groups compared with the other groups (Fig. 5A–D). We quantified NETs by PicoGreen, and the results showed that the release of NETs was increased in the IDH1-K93R and MDH1-K118R groups compared with the other groups. However, ACY1215 reduced NETs release (Fig. 5E). Consistently, we observed attenuated histopathological damage in the ACY1215 group, substantial death of hepatocytes, and significantly increased numbers of infiltrating inflammatory cells in the IDH1-K93R or MDH1-K118R group (Fig. 5F). We believe that IDH1/MDH1 deacetylation aggravates LPS/d-gal-induced liver injury in mice by enhancing the formation of NETs. Interestingly, TUNEL staining in mouse liver tissue showed increased apoptosis in the IDH1-K93R or MDH1-K118R group compared with the other groups (Fig. 5G).

## Discussion

NETosis is a double-edged sword [7]. NETs participate in the body’s defense mechanism and are released as a physical barrier to the outside of cells to capture and kill pathogens and limit the dissemination of inflammatory mediators. This process protects the normal tissues of the body from destruction [6, 26]. However, when the production of NETs is abnormally regulated, the beneficial defense mechanism is transformed into a harmful mechanism that leads to NETs-related damage in the body [26]. As a heterogeneous and fatal disease, ALF is characterized by acute onset and high mortality. This study confirms the presence of NETs in the liver tissue of patients with ALF. Although the formation of NETs has been found in various liver diseases, including nonalcoholic steatohepatitis [27], the mechanism by which NETosis regulates the development of ALF remains unknown. A better understanding of the mechanisms of NETosis may lead to new approaches for the treatment of ALF.

Posttranslational modifications (PTMs), including acetylation, phosphorylation, and methylation, play key roles in various biological processes by altering protein structure and dynamics [28, 29]. PTMs can occur in single or multiple amino acids and result in changes in the chemistry of the modification site [30]. Isocitrate dehydrogenase (IDH) is an essential metabolic enzyme involved in the TCA cycle. There are three isoforms of IDH [31], among which IDH1 is located in the cytoplasm and peroxisomes and catalyses the reversible oxidative decarboxylation of isocitrate to α-ketoglutarate (α-KG) [32, 33]. During this process, nicotinamide adenine dinucleotide phosphate (NADP^+^) is reduced to reduced nicotinamide adenine dinucleotide phosphate (NADPH) [32, 33]. IDH1 is the major producer of NADPH in most tissues [33]. Thus, IDH1 indirectly attenuates oxidative damage by regulating NADPH and α-KG [33, 34]. Studies have shown that when a single amino acid change occurs in a conserved arginine residue in the isocitrate-binding site of IDH1, it will lead to a decrease in the enzymatic activity of the oxidative decarboxylation of isocitrate to α-ketoglutarate [35]. In addition, it has been shown that acetylation may be an important posttranslational modification that regulates the catalytic efficiency of IDH1 and is associated with IDH1 activity [36].

Malate dehydrogenase 1 (MDH1), which reversibly catalyses the oxidation of malate to oxaloacetate, is one of the key enzymes in glucose metabolism [37]. This molecule participates in the malate-aspartate shuttle, coordinates glycolysis and mitochondrial respiration, and plays an important role in oxidative stress. In this process, the coenzyme II (NADPH)/oxidized coenzyme (NADP^+^) ratio and ROS activity are reduced [37, 38]. Studies have shown that MDH1 acetylation acts as a negative regulator of oxidative stress. Oxidative stress is enhanced when MDH1 expression or activity is reduced. Posttranslational acetylation can increase MDH1 activity and reduce oxidative stress. Researchers confirmed that the increase in MDH1 activity after acetylation was achieved by increasing the production of acetyl-CoA and NADPH to promote fatty acid synthesis [37]. Thus, acetylation of MDH1 activates its enzymatic activity and increases intracellular NADPH levels, thereby regulating adipogenic differentiation [37, 39].

Our previous studies have shown that the occurrence of ALF is accompanied by energy metabolic disorders. In an ALF mouse model, HDAC6i alleviated liver pathological changes and improved liver function [16]. Moreover, HDAC6i was shown to modify IDH1 lysine 93 and MDH1 lysine 118 through acetylation in the tricarboxylic acid cycle. This process promotes energy metabolism to relieve ALF. In this study, we found that IDH1-K93 and MDH1-K118 mutations deacetylated IDH1 and MDH1 in neutrophil-like cells (dHL-60), while significantly enhancing the formation of NETs after PMA induction. In ALF mice infected with IDH1-K93R and MDH1-K118R adenoviruses, the formation of NETs in liver tissue and serum was greater than that of ALF model group mice, extensive liver cell death was observed, and the number of inflammatory cells increased. Thus, IDH1/MDH1 deacetylation promotes NETosis and accelerates the progression of ALF.

Therefore, in the process of ALF, energy metabolism is disturbed by affecting the important metabolic enzyme IDH1/MDH1 in the process of glucose metabolism. Further studies found that IDH1/MDH1 deacetylation affects the occurrence of “suicidal” NETosis, which promotes ALF. Combined with other studies, we believe that deacetylation of IDH1/MDH1 enhances oxidative stress and increases ROS activity. This process makes histone-modifying enzymes, such as NE and PAD4, enter the nucleus. It promotes the unfolding of chromatin, the rupture of the nuclear envelope, the release of chromatin, granule proteins and other components outside the cell, and the eventual formation and release of NETs. ALF is exacerbated by local tissue damage produced by NETs.

In this study, an LPS/d-gal-induced ALF mouse model was used to mimic the etiology of patients with ALF. Moreover, neutrophils are terminal cells with a short survival time, which was addressed by inducing the differentiation of HL-60 cells. During the study, we found that HL-60 cells with fewer passages were more likely to release NETs after PMA induction.

Apoptosis is a typical pathological feature of liver diseases. Interestingly, increased hepatocyte apoptosis was observed during ALF with increased NETs release. Therefore, we speculate that various modes of cell death may not be independent in ALF. They may occur simultaneously or crosstalk with each other. Activation or inhibition of one mode of cell death may activate other modes of cell death. It may be the reason why drugs that only inhibit a certain cell death mode are not effective in treating acute liver failure. This issue should be studied in the future.

## Conclusions

IDH1-K93 and MDH1-K118 mutations reduce acetylation of IDH1 and MDH1, which promotes the formation of NETs and aggravates the progression of acute liver failure.

## Data Availability

All data are contained within this article.

## Abbreviations

IDH1: Isocitrate dehydrogenase 1
MDH1: Malate dehydrogenase 1
ALF: Acute liver failure
PMA: Phorbol myristate acetate;
LPS: Lipopolysaccharide
NETs: Neutrophil extracellular traps
PKC: Protein kinase C
NOX: NADPH oxidase complex
ROS: Oxygen species
PAD4: Peptidylarginine deiminase 4
CitH3: Citrullinated histone H3
MPO: Myeloperoxidase
NE: Neutrophil elastase
PLT: Platelets
SK3: Small conductance potassium channel member 3
TCA: Tricarboxylic acid
HDAC6i: Histone deacetylase inhibitor 6
dHL-60: Differentiated HL-60 cells
d-gal: d-galactosamine
PLL: Poly-l-Lysine
DAPI: 4′,6-Diamidino-2-phenylindole
TUNEL: Terminal deoxynucleotidyl transferase dUTP nick-end labeling
PTMs: Posttranslational modifications
NADPH: Nicotinamide adenine dinucleotide phosphate
